# Climate changes modulated the history of Arctic iodine during the Last Glacial Cycle

**DOI:** 10.1038/s41467-021-27642-5

**Published:** 2022-01-10

**Authors:** Juan Pablo Corella, Niccolo Maffezzoli, Andrea Spolaor, Paul Vallelonga, Carlos A. Cuevas, Federico Scoto, Juliane Müller, Bo Vinther, Helle A. Kjær, Giulio Cozzi, Ross Edwards, Carlo Barbante, Alfonso Saiz-Lopez

**Affiliations:** 1 Department of Atmospheric Chemistry and Climate, Institute of Physical Chemistry Rocasolano, CSIC, Serrano 119, 28006 Madrid, Spain; 2 Physics of Ice Climate and Earth, Niels Bohr Institute, University of Copenhagen, Tagensvej 16, Copenhagen N, 2200 Denmark; 3 Institute of Polar Sciences, CNR- ISP, Via Torino 155, 30172 Venice, Italy; 4 Ca’ Foscari University of Venice, Department of Environmental Sciences, Informatics and Statistics, Via Torino 155, 30172 Venice, Italy; 5 Institute of Atmospheric Sciences and Climate, ISAC-CNR, S.P Lecce-Monteroni km1.2, 73100 Lecce, Italy; 6 Alfred Wegener Institute, Helmholtz Center for Polar and Marine Research, Am Alten Hafen 26, 27568 Bremerhaven, Germany; 7 MARUM Research Faculty, University of Bremen, Leobener Strasse 8, 28359 Bremen, Germany; 8 Physics and Astronomy, Curtin University, Kent St, Bentley, WA 6102 Australia; 9 Department of Civil and Environmental Engineering, UW-Madison, Madison, WI 53706 USA; 10 Present Address: CIEMAT, Environmental Department, Av. Complutense 40, 28040 Madrid, Spain

**Keywords:** Climate sciences, Palaeoclimate

## Abstract

Iodine has a significant impact on promoting the formation of new ultrafine aerosol particles and accelerating tropospheric ozone loss, thereby affecting radiative forcing and climate. Therefore, understanding the long-term natural evolution of iodine, and its coupling with climate variability, is key to adequately assess its effect on climate on centennial to millennial timescales. Here, using two Greenland ice cores (NEEM and RECAP), we report the Arctic iodine variability during the last 127,000 years. We find the highest and lowest iodine levels recorded during interglacial and glacial periods, respectively, modulated by ocean bioproductivity and sea ice dynamics. Our sub-decadal resolution measurements reveal that high frequency iodine emission variability occurred in pace with Dansgaard/Oeschger events, highlighting the rapid Arctic ocean-ice-atmosphere iodine exchange response to abrupt climate changes. Finally, we discuss if iodine levels during past warmer-than-present climate phases can serve as analogues of future scenarios under an expected ice-free Arctic Ocean. We argue that the combination of natural biogenic ocean iodine release (boosted by ongoing Arctic warming and sea ice retreat) and anthropogenic ozone-induced iodine emissions may lead to a near future scenario with the highest iodine levels of the last 127,000 years.

## Abstract (web summary)

Iodine is important for new particle formation in the atmosphere, but how it varies over long time scales is not well known. Here, the authors present ice core data from the last 127,000 years that show that iodine varied between glacials and interglacials, but also showed abrupt changes in pace with sea-ice and temperatures.

## Introduction

Atmospheric iodine, primarily emitted from oceans, forms new aerosol particles in the atmosphere^1–7^ and efficiently destroys ozone in the troposphere and lower stratosphere^8–11^, which reduces ozone radiative forcing^12–15^. In the Arctic environment, iodine has recently been identified as a significant source of cloud condensation nucleii (CCN), with the potential to influence clouds formation^3^. Iodine in the atmosphere is increasing globally (3-fold since the 1950s), as independently evidenced by polar and alpine ice core and tree ring measurements, following anthropogenic ozone pollution and global warming^16–18^. In the Arctic, a coastal ice core from Greenland revealed that ocean primary productivity controlled atmospheric iodine variability during the Holocene (i.e., last 11,700 years)^19^. Before the climatically stable Holocene, the Arctic underwent abrupt environmental changes featuring vast ice sheet growth and marked climate events during the Last Glacial Period (LGP) (115–11.7 kyr before 2000 CE (b2k)) and experienced temperatures warmer than today during the Last Interglacial Period (Eemian, 130–115 kyr b2k). However, to date, the natural evolution of Arctic iodine before the Holocene, in the absence of anthropogenic forcings, and its coupling with climate variability remain unknown.

**Fig. 1:**
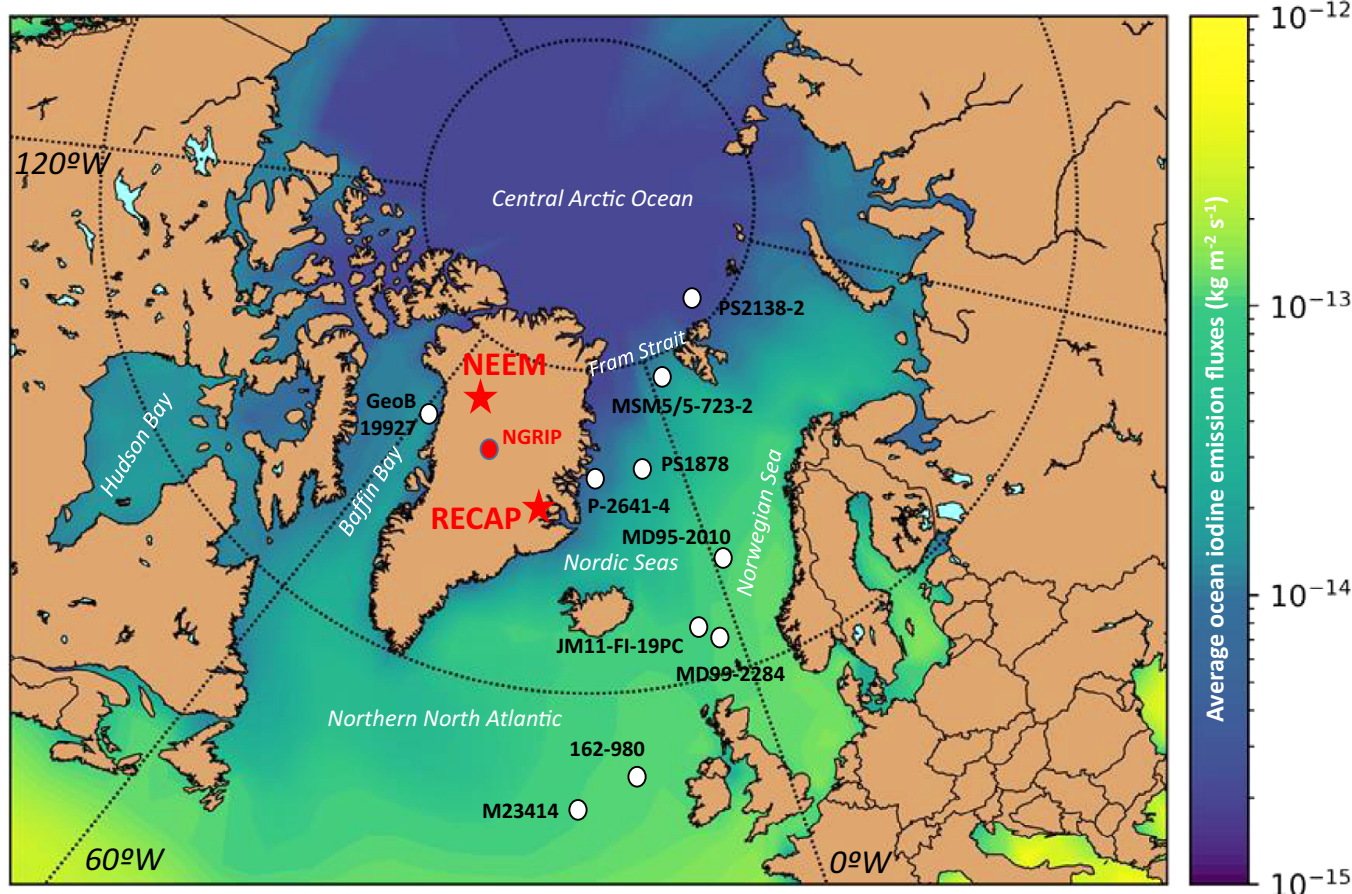
Present-day (CE 2014) total iodine emission fluxes from the oceanic regions influencing Greenland. Modelled mean annual total iodine emission fluxes (CH_3_I + CH_2_I_2_ + CH_2_IBr + CH_2_ICl + HOI + I_2_) from the North Atlantic and Arctic Oceans using the 3D chemistry-climate model CAM-Chem. The figure includes the location of the NEEM and RECAP ice cores (red) and other ice cores and marine paleoceanographic archives in the Arctic and the northern North Atlantic discussed in the text and figures.

In this work, we report the iodine levels in the Arctic during the Last Glacial Cycle (LGC) (last 127 kyr) using records from two Greenland ice cores (NEEM and RECAP) drilled in northwestern and eastern coastal Greenland, respectively (Fig. 1). Both ice cores provide continuous paleoenvironmental records back to the Eemian interglacial period^20–22^. We provide evidence that ocean-ice-atmosphere exchange of biogenic iodine in the Arctic Ocean dominated the atmospheric iodine budget during periods of rapid climatic changes. The results show that abrupt warming and sea ice retreat in the Arctic preceded maximum iodine levels, reaching peak concentrations during interglacial periods.

## Results and discussion

### Present-day iodine emission sources and depositional fluxes over Greenland

Atmospheric iodine in Greenland is controlled by a complex interplay involving iodine emissions from the oceans, as well as particle-bound iodine compounds related to dust and/or sea spray aerosol (ssa) variability^23^. The oceans, which are the largest reservoirs of iodine on Earth^24^, are known to be the dominant source of atmospheric iodine^25–27^. The major oceanic sources of gas-phase iodine are (i) inorganic, ozone-induced, hypoiodous acid and molecular iodine emissions and (ii) organic iodine from the metabolic activity of primary producers (phytoplankton and macro- and micro-algae). Furthermore, atmospheric iodine can also be related to mineral dust by promoting iodate stability in the ice matrix and/or favouring the adsorption of iodine gas-phase molecules to dust particles during atmospheric transport^10^. On the other hand, ssa expelled from the ocean surface during wave breaking incorporate iodine in their composition and are a substrate where gaseous iodine species undergo heterogeneous recycling reactions^28–30^.

We used a global chemistry climate model^17^ to understand present-day iodine levels in Greenland (see Methods). Present iodine depositional fluxes at the RECAP drilling site (71°18′ N; 26°43′ W; 2315 m a.s.l.) are ~51% higher than at NEEM (77°45′ N, 51°06′ W; 2484 m a.s.l.) (Supplementary Fig. 1). This is explained by the different ocean iodine emission strengths at the source regions of air masses that influence both sites (the Canadian Arctic, Baffin Bay and Hudson Bay for NEEM^31^ and the North Atlantic and Arctic Ocean for RECAP^32^). Note also that RECAP is closer to the coast (30 km from the North Atlantic Ocean) than NEEM (350 kms from the Baffin Bay). Our model results show higher iodine emissions in the RECAP source regions (50N–80N, 45W–12E; mean iodine emissions of 9.1 × 10^−14^ kg m^−2^ s^−1^) than in the NEEM source areas (50N–80N, 120W–45E; mean iodine emissions of 3.5 × 10^−14^ kg m^−2^ s^−1^) (Fig. 1).

### The Greenland iodine records

Total iodine and sodium ([I], [Na]) concentrations from NEEM and RECAP ice core samples were determined at 1.10 m and 0.55 m mean intervals of melted ice, respectively (Supplementary Figs. 2-4). Calcium ([Ca]) concentrations were also used as proxies of mineral dust in Greenland ice cores^33,34^. These geochemical measurements along with other ice cores and marine paleoceanographic archives allow the reconstruction of Arctic iodine variability and environmental sources during the LGC.

**Fig. 2:**
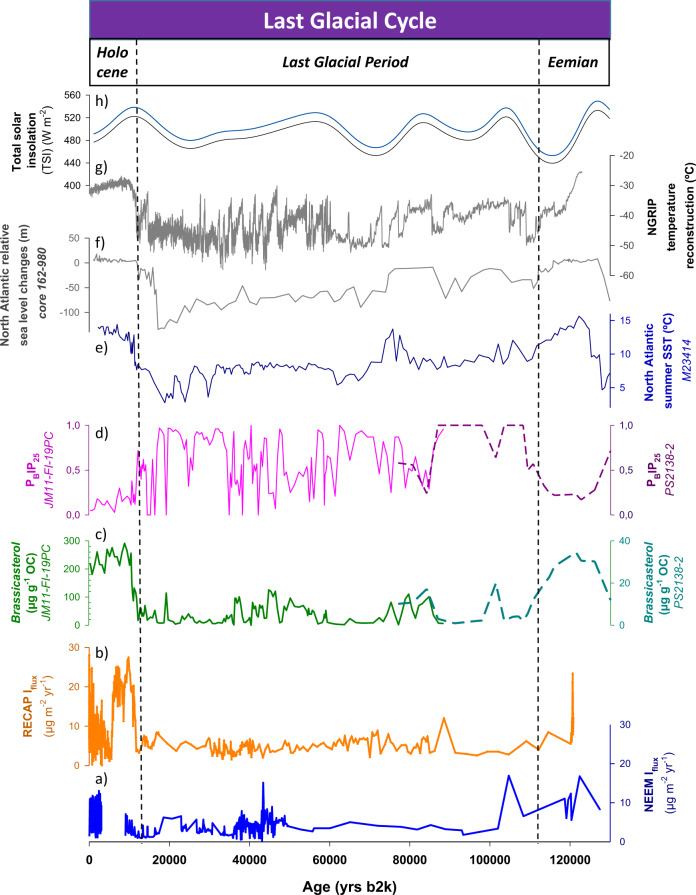
Iodine levels measured in the NEEM and RECAP ice cores together with other paleoenvironmental proxies for the last 130 kyr b2k. From bottom to top: **a**, **b** iodine depositional fluxes (I_flux_) from NEEM and RECAP ice cores; **c**, **d** Arctic primary productivity and sea ice evolution (*brassicasterol* and P_B_PIP_25_ profiles, respectively^38,46^); **e** North Atlantic summer sea surface temperature (SST)^36^; **f** North Atlantic relative sea level changes^88^; **g** Greenland air temperature reconstruction from NGRIP^64,65^; **h** solar irradiation reconstruction in July at 77° (NEEM) and 71°N (RECAP)^89^.

Maximum iodine levels were found during the Eemian in the NEEM ice core, with a total mean iodine depositional flux (I_flux_) of 9.6 µg m^−2^ yr^−1^ coinciding with the highest Arctic Ocean primary productivity inferred from the phytoplankton biomarker *brassicasterol*^35^ (Fig. 2). Our analysis suggests that these high Eemian iodine levels resulted from the interplay of (i) enhanced biogenic iodine emissions driven by primary productivity; (ii) higher sea surface temperatures (SSTs) in the Arctic and the North Atlantic^36,37^, which facilitates sea-air transfer of volatiles; and (iii) dominant open water conditions in the Arctic, as reflected by the low P_B_IP_25_ values. P_B_IP_25_ has been used as a proxy of the intensity of sea ice cover in the Arctic^38^, as well as in Baffin Bay^31,39^ since it accounts for the algal and phytoplankton (*brassicasterol*) biomarkers activity, which allows to assess the spatial and temporal extent of the sea ice cover^40^ (Fig. 2). The Arctic sea ice extent was significantly reduced during the Eemian, and minimum sea ice concentrations towards almost ice-free summers occurred in wide areas as far north as the northern Barents Sea as a consequence of the strong inflow of warm Atlantic waters^38^, thus facilitating springtime blooms and oceanic iodine production. Indeed, summer North Atlantic SSTs during the Eemian were 2 °C greater than at present^36,37^. Furthermore, a mean global sea level 6–8 m above the present level^41–43^ (Fig. 2) triggered the flooding of most parts of the shallow Siberian marginal seas, eventually resulting in more productive coastal areas in the Arctic. At NEEM, the highest I_flux_ was recorded at ~122 kyr b2k concomitant with maximum levels of biological productivity, sea level rise and SST. The coupling of all these drivers during the Eemian would have eventually released large amounts of iodine into the Arctic atmosphere, resulting in the largest iodine emissions on record. The significant decrease in NEEM and RECAP I_flux_ recorded during the last glacial inception at ~120 kyr b2k is correlated with an increase in sea ice^31,38^ and a decrease in both SST and *brassicasterol* (Fig. 2), as also indicated by independent modelling experiments that resemble pre-industrial scenarios^41^.

**Fig. 3:**
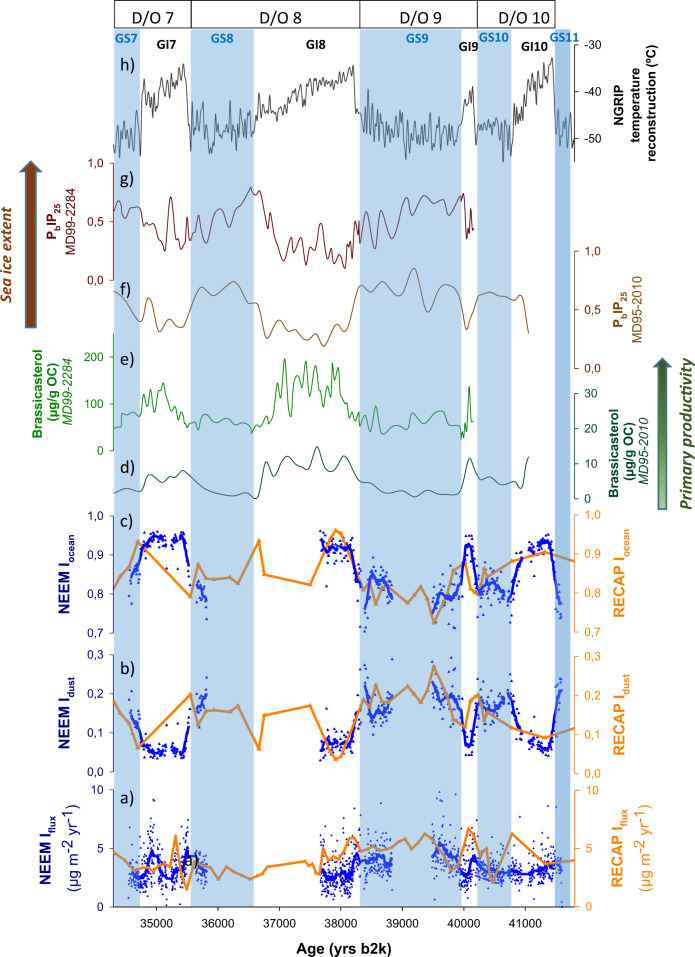
High-resolution iodine level variability in Greenland NEEM and RECAP ice cores from 34 to 42 kyr b2k (=D/O 7 to 10). From bottom to top: **a** high-resolution NEEM (dark yellow) and RECAP (red) iodine depositional fluxes (I_flux_), **b**, **c** dust-related iodine (I_dust_) and reconstructed ocean iodine emissions (I_ocean_), with dark yellow lines representing the 11-sample running average in NEEM; **d**, **e** Arctic primary productivity and **f**, **g** sea ice evolution (*brassicasterol* and P_B_PIP_25_ profiles, respectively) from sediment cores in the Norwegian Sea^48,49^; **h** Greenland air temperature reconstruction from NGRIP^64^. Blue and white bands represent Greenland interstadials (GIs) and stadials (GSs), respectively.

Ice core iodine showed reduced values throughout the LGP, with mean NEEM and RECAP I_flux_ of 3.8 and 5.1 µg m^−2^ yr^−1^, respectively (Fig. 2). The LGP is characterized by abrupt climatic variability marked by Dansgaard/Oeschger (D/O) events. These climatic phases consist of warm Greenland interstadials and cold Greenland stadials that are well recorded in Greenland ice cores and marine sediments from the North Atlantic region and the Nordic seas^44–49^. To investigate in detail the iodine variability across the rapid D/O transitions, additional high-resolution iodine measurements were carried out in the NEEM ice core from 34 to 42 kyr b2k (D/O 7 to 10), with a 3- to 10-year resolution (Fig. 3 and Supplementary Fig. 4). In order to differentiate the main sources of iodine that determine I_flux_ variability, we have decoupled oceanic iodine emissions (I_ocean_) from dust-related iodine (I_dust_) (‘Methods’). The sub-decadal resolution measurements of iodine during this period show the concomitant evolution of I_ocean_, Arctic Ocean primary productivity and sea ice variability^46,48,49^ (Fig. 3). Each D/O event showed a similar sequence: (i) iodine emissions increased concurrently with a reduction in sea ice at the onset of the interstadials; (ii) iodine emissions decreased during the late interstadial periods as the climate cooled and sea ice gradually increased; and iii) negligible iodine was released from the oceans with the development of thicker sea ice conditions during the cold stadial periods.

Interestingly, NEEM and RECAP I_flux_ and I_ocean_ do not follow the same trend during D/O events (Fig. 3). In the context of strongly reduced iodine emissions during the LGP due to the extensive development of perennial sea ice in the Canadian Arctic^31^, a significant fraction of iodine registered in the NEEM ice core was strongly controlled by dust and, to a lesser extent, by ssa. This is corroborated by the robust statistical correlations between [I] and [Ca] and between [I] and [Na] from D/O 7–10 (*r* > 0.75 and 0.78, respectively). These correlations are also seen in RECAP during the LGP (*r* > 0.88 and 0.87, respectively). Sea spray aerosol emissions (indicated by [Na] concentrations in both ice cores) show a 50% increase during the LGP^33^ (Supplementary Figs. 2 and 3), thereby contributing to higher deposition of ssa-related iodine during this period. On the other hand, [Ca] concentrations in Greenland ice cores show a major increase during stadials^33^ (Supplementary Figs. 2 and 3) due to enhanced mineral dust associated with increased aridity in East Asia and enhanced atmospheric transport mechanisms^33,50,51^. The higher I_dust_ values found in NEEM and RECAP during the coldest phases of the LGP are in agreement with the highest iodine fluxes recorded in the Talos Dome ice core (eastern Antarctica) between 16.8 and 33.8 kyr b2k^10^. This Antarctic ice core reported a correlation between iodate, the most stable iodine species in the atmosphere, and atmospheric dust, indicating uptake of gas-phase iodine molecules into fine dust particles during atmospheric transport^10^.

**Fig. 4:**
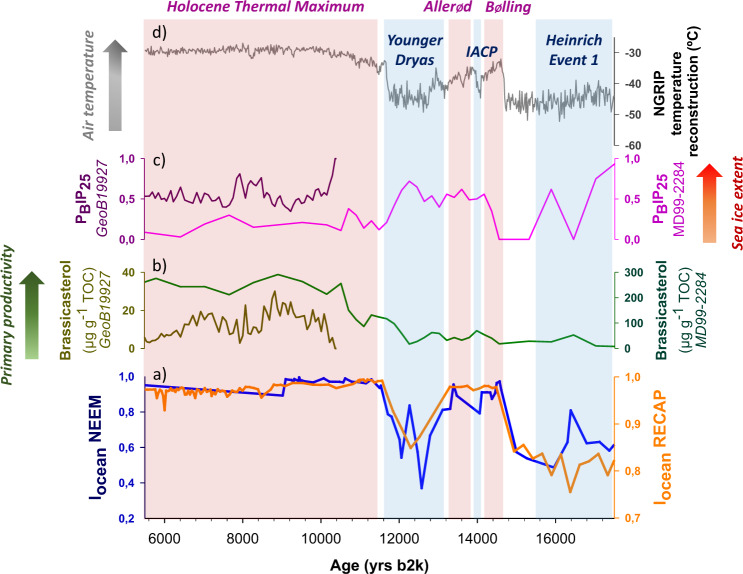
I_ocean_ variability in Greenland ice cores during the Last Glacial Termination/Holocene transition. From bottom to top: **a** I_ocean_ values in NEEM (dark yellow) and RECAP (red) ice cores; **b**, **c** Arctic primary productivity and sea ice evolution (*brassicasterol* and P_B_PIP_25_ profiles, respectively) in Baffin Bay (core GeoB19927)^60^ and the Norwegian Sea (core MD99-2284)^46^; **d** Greenland air temperature reconstruction from NGRIP^64^. Bands indicate the main cold (blue) and warm (red) climatic phases of this period (IACP = Intra-Allerød Cold Period).

The largest iodine variability occurred during the LGP-Holocene transition when iodine emissions strongly fluctuated following the main climatic phases of the Last Glacial Termination. NEEM and RECAP I_ocean_ showed very similar evolution patterns, with minimum I_ocean_ values during Heinrich event 1 (H1, 16.8 kyr b2k) (Fig. 4), when surface waters cooled and a thick perennial sea ice cover reached both the Norwegian Sea and Baffin Bay^31,46,52^. The I_ocean_ increased in both ice cores at the onset of the Bølling (14.7–14.1 kyr b2k) warming in the Arctic, slightly decreased during the Intra-Allerød Cold Period (14.1–13.9 kyr b2k) and increased again during the Allerød (13.9–12.9 kyr b2k) warm period (Fig. 4). The increase in I_ocean_ values during the Bølling-Allerød period was synchronous with an abrupt increase in ocean productivity as far north as the Fram Strait^53^. NEEM and RECAP I_ocean_ moderately decreased during the last cold spell of the Last Glacial Termination, the Younger Dryas (12.9–11.7 kyr b2k).

Oceanic iodine emissions increased in both ice cores at the onset of the Holocene (~11 kyr b2k; Fig. 4) resulting from the interplay of different factors: (i) enhanced marine primary productivity in the Arctic^46,54–56^; (ii) dominant open water conditions in the subpolar North Atlantic^46^; (iii) abrupt sea level rise (~120 m) during the Last Glacial Termination^57,58^ resulting in more shallow oceanic domains increasing iodine emission from coastal areas; and iv) maximum levels of solar irradiance in the Arctic that would have also increased oxidative stress in algae, leading to an enhancement of biological iodine production in the ocean and subsequent release into the Arctic atmosphere^59^. I_flux_ strongly increased in RECAP from 11 to 9 kyrs b2k, while the NEEM I_flux_ increase was significantly more gradual at that time (Fig. 2), most likely due to the lower productivity and delayed sea ice retreat in Baffin Bay during the early Holocene^60^ (Fig. 4). Indeed, this area remained densely sea ice covered until ~7.4 kyr b2k, when warmer North Atlantic waters penetrated Baffin Bay, increasing regional bioproductivity^61^ (Fig. 4). The mean Holocene iodine flux in NEEM is 57.9% of that in RECAP (mean iodine fluxes of 5.1 and 8.8 µg m^−2^ yr^-1^, respectively), in agreement with present-day reactive iodine deposition at both sites obtained from atmospheric chemistry transport model results (Supplementary Fig. 1).

### Environmental implications

Ocean-emitted iodine leads to new particle formation (NPF) in the atmosphere^5,6^. Recent field observations have demonstrated that iodine is a significant driver of NPF in the central Arctic^3^ and is currently considered a potentially relevant source of CCN^3^—from which marine clouds originate—that scatter incoming radiation and can contribute a cooling effect to the Earth´s radiation budget^62^. The significant present-day contribution of iodine to NPF leads us to speculate that, in the absence of other human-induced NPF nucleation drivers during pre-industrial times^63^, oceanic iodine may have played an even larger relative role in total NPF nucleation in a pristine Arctic during the LGC.

We find that the time lag between temperature changes and iodine maximum emission values recorded in Greenland ice cores throughout the LGC strongly depends on the atmosphere-ice-ocean interactions driving warming SST, sea ice thinning rate and algae metabolic activity that eventually control biogenic iodine emissions. Indeed, the highest iodine emission periods during the LGC were preceded by the highest air temperatures that would have first enhanced SST and ocean biological activity and later favoured the transfer of volatile iodine compounds from the ocean to the atmosphere. Together with the efficiency of iodine to destroy ozone and activate CCN in the polar atmosphere, this points to a possible contribution of iodine to ozone loss and new particle formation, and to their associated radiative impacts, during past abrupt climate change periods, such as D/O events and the onset of interglacials. However, this potential contribution of iodine to past ozone loss and atmospheric NPF is currently unknown and warrants further research.

We now turn to the use of past warm intervals recorded in Greenland ice cores as analogues to evaluate potential feedback and thresholds in the Arctic climate system and their implications for future warming impacts. The rate of ongoing atmospheric warming resembles only the abrupt climate changes and sea ice retreat that occurred during the D/O events^64^, with climate warming of 5–16.5 °C occurring within a century^65^. The high resolution NEEM iodine dataset during D/O 7–10 show empirical evidence of a fast response of iodine emissions synchronously with the rapid North Atlantic sea ice retreat preceding abrupt Greenland warming during D/O events^48^. Therefore, one question arising is if Arctic iodine levels are facing this scenario in the near future?

Iodine emissions and depositional fluxes during the Eemian and/or Holocene Thermal Maximum shed light on the possible environmental conditions in an expected ice-free Arctic Ocean and thus remain useful as an observational constraint on projections of future impacts. Thus, while atmospheric iodine depositional fluxes recorded in RECAP during the Holocene Thermal Maximum^19^ mirror present-day conditions^17^, iodine levels recorded in NEEM during the mid-Eemian tripled present-day values at this location. Similar trends might be expected in near future environmental scenarios including (i) predicted ice-free summertime conditions in the Arctic Ocean by 2050 CE^66^ and (ii) 3–4 °C warmer global temperatures with a three- to four-fold amplification in the Arctic by 2100 CE^67^. However, one important point to consider is that the predicted iodine emissions in the Arctic in the near future based on past analogues represent only the biogenic fraction of the total foreseen atmospheric iodine concentrations in the Arctic. Ozone-induced inorganic iodine emissions during these past warm periods could be considered negligible in the context of the very low ozone concentrations before industrialization^19,68^. In contrast, at present, ocean release of inorganic iodine is estimated to account for 75% of the total source of atmospheric iodine, with the remaining 25% coming from biogenic emissions^69^. Furthermore, ozone-related inorganic iodine emissions are predicted to increase during the 21st century^15^. Therefore, we argue that the future combined (inorganic + biogenic) ice-free Arctic Ocean iodine emissions might not have analogues in the past, which could potentially lead to a near future scenario with the highest iodine levels of the last 127,000 years.

In summary, this study uncovers how past climate changes modulated the variability of the ocean-atmosphere exchange of iodine in the Arctic during the LGC. We show that ocean productivity was the dominant source of Arctic iodine during interglacial periods, leading to highest levels during the Holocene and the Eemian, while dust-related iodine constituted a significant source of iodine in the Arctic atmosphere during the coldest phases of the Last Glacial Period. The mirrored trends of iodine emissions and ocean primary productivity proxies during different past environmental scenarios highlight the key role of ocean biology and sea ice cover in driving iodine variability in the Arctic region. Finally, we conclude that understanding the past variability of iodine as a key environmental element is important to further comprehending the evolution of Arctic atmospheric chemistry on centennial to millennial timescales and its coupling to climate changes.

## Methods

### Ice core chronologies and sampling resolution

NEEM age-depth model was achieved by transferring the annual layer counting from the NGRIP to the NEEM ice cores using 787 tie points (mainly volcanic layers)^21^. NEEM samples were collected at 1.10 m mean intervals of melted ice where 10 mL of meltwater was collected in an acid-cleaned polyethylene bottle and immediately frozen. Chronological resolution between samples (*n* = 84) ranges from 4 to 267 years during the Holocene. There is a sampling gap between 3-9 ky b2k where the ice was not analysed due to the poor quality of the core in the “brittle ice” section^31^. The mean sampling frequency during the Glacial Period (n = 247) is ~400 years, where the highest resolution achieved was between 25 and 50 kyrs b2k with sampling resolution ranging from 60 to 150 years during warmer interstadial periods and colder glacial stadial periods, respectively. Additional sampling was carried out during the intervals 34.5–35.8, 37.6–38.8 and 39.4–41.6 (*n* = 1304) providing a time resolution ranging from 3 to 10 years during those intervals to better resolve the rapid stadial-interstadial transition.

RECAP age-depth model is based on (i) annual layer counting from the last four millennia using the StratiCounter algorithm^70^ constrained by volcanic eruption markers and synchronized to the GICC05 Greenland Ice Core Chronology framework^71^; (ii) a modified Dansgaard-Johnsen ice flow model^72^ constrained to well-dated age markers from 4048 to 11,703 ka b2k and (ii) linear interpolation between 73 GICC05-modelext age markers between 11,703 and 120,215 years b2k^32^. Each RECAP sample integrated a 55 cm depth interval, where the resolution in the Holocene (*n* = 1035) ranged from sub-annual in the upper metres to decadal or centennial resolution during the mid to early Holocene (average resolution of 12 years per sample). Due to the thinning of annual layers according to depth, each sample integrates approximately 470 years during the glacial period (*n* = 218)^32^. For details on the age model we refer the readers to Simonsen et al.^22^.

### Geochemical analyses

Samples from NEEM and RECAP ice cores were collected using continuous flow analysis systems. NEEM samples were sent to the Environmental Analytical Chemistry laboratory of the CNR-ISP and Ca’ Foscari University of Venice for iodine and sodium analyses. Total iodine and sodium concentrations were determined by inductively coupled plasma mass spectrometry (ICP-MS). Sodium and iodine detection limits were 1 ppb and 0.005 ppb, respectively. RECAP samples were sent to ISP-CNR and to Curtin University of Technology (Perth, Australia). Total iodine and sodium concentrations in Perth were determined by inductively coupled plasma-sector field mass spectrometry (ICP-SFMS) with sodium and iodine detection limits of 1.1 ppb and 0.002 ppb, respectively. The analytical procedure for both records was performed following Vallelonga et al.^73^ and Maffezzoli et al.^32^. Instrumental errors for iodine and sodium concentrations were 5%.

### I_flux,_ I_dust_ and I_ocean_ calculation

Total iodine depositional fluxes (I_flux_) in the ice cores were calculated following Corella et al.^19^. To estimate I_flux_ in the RECAP ice core, we re-scaled NGRIP accumulation rates^65^ by a factor of 436/174 since it is the modern accumulation ratio at the NGRIP and RECAP drilling sites^74–76^. Dust-related iodine (I_dust_) and oceanic gas-phase iodine emissions (I_ocean_) were calculated according to Eqs. (1) and (2)

1 $${{{{{{\rm{I}}}}}}_{{{{{\rm{dust}}}}}}}={{{{{\rm{nssCa}}}}}}\,{{{{{\rm{X}}}}}}([{{{{{\rm{I}}}}}}]{/}[{{{{{\rm{Ca}}}}}}])_{\left.{{{{{\rm{crust}}}}}}\right)}{/}[{{{{{\rm{I}}}}}}]$$

2 $${{{{{{\rm{I}}}}}}_{{{{{\rm{ocean}}}}}}}=({{{{{\rm{nssI}}}}}}-({{{{{\rm{nssCa}}}}}}\,{{{{{\rm{X}}}}}}{([{{{{{\rm{I}}}}}}]{/}[{{{{{\rm{Ca}}}}}}])}_{{{{{{\rm{crust}}}}}}})){/}[{{{{{\rm{I}}}}}}]$$

with non-sea salt iodine (nssI) representing the excess iodine production beyond the iodine production related to ssa. It is calculated as nssI = [I] − [Na] × ([I]/[Na])_seawater_ with a [I]/[Na] seawater concentration ratio of 5.6 × 10^−6^ ^77^. Non-sea salt calcium (nssCa) represents the non-sea spray aerosol fraction of calcium and is representative of mineral dust input. It is calculated as nssCa = [Ca] − ssCa, where ssCa = [Na] × ([Ca]/[Na])_seawater_ with a [Ca]/[Na] seawater concentration ratio of 0.0391. ([I]/[Ca])_crust_ represents the ratio of both elements in the continental crust (4.75 × 10^−5^ ^78^). Both, I_ocean_ and I_dust_ correspond to unitless fractional proxies.

### Atmospheric chemistry modelling

We also used the global 3-D chemistry-climate Community Atmospheric Model with chemistry (CAM-Chem) version 4 to estimate (i) oceanic iodine emissions from different source areas in the Arctic and North Atlantic Oceans (Fig. 1) and (ii) the deposition fluxes of reactive iodine (HI + HOI + I_2_O_2_ + I_2_O_3_ + I_2_O_4_ + INO_2_ + IONO_2_) in Greenland (Supplementary Fig. 1). CAM-Chem includes a comprehensive benchmark chemistry scheme to simulate the evolution of trace gases and aerosols in the troposphere and stratosphere^79,80^. The model implements a halogen chemistry scheme for chlorine, bromine and iodine^12,81,82^. This includes the photochemical breakdown of five very short-lived bromocarbons (VSL^Br^ = CHBr_3_, CH_2_Br_2_, CH_2_BrCl, CHBrCl_2_, CHBr_2_Cl) and four iodocarbons (VSL^I^ = CH_3_I, CH_2_ICl, CH_2_IBr, CH_2_I_2_), which are naturally emitted from the ocean into the atmosphere^83^. Additionally, abiotic oceanic sources of HOI and I_2_ were included in the lowest layer of the model^26^ based on laboratory studies of the oxidation of aqueous iodide by atmospheric ozone deposited on the ocean surface^25,84^. In this simulation, the model was configured with a horizontal resolution of 1.9° latitude by 2.5° longitude and 26 levels, from the surface to ∼40 km (with eight levels above 100 hPa)^17,29,82^. At the lower boundary, the time-varying zonally averaged distributions of CO_2_, CH_4_, H_2_, N_2_O and long-lived halocarbons (CFC-11, CFC-12, CFC-113, HCFC-22, H-1211, H-1301, CCl_4_, CH_3_CCl_3_, CH_3_Cl and CH_3_Br) are specified following their observed distribution for 2000 CE^85^. Monthly mean time variations observed for sea surface temperature and sea ice distribution are also prescribed. To obtain a reasonable representation of the overall stratospheric circulation, the integrated momentum that would have been deposited above the model top is specified by an upper boundary condition. We used the output of a previous work^17^ for CE 2010. This simulation was run in free-running mode^79^ with prescribed sea surface temperatures, sea ice and meteorological fields from 1950 to 2010^86^. The simulated dynamics and transport therefore represent the daily synoptic conditions of the observations in CE 2010, allowing the online coupling between the ocean, ice and atmospheric modules of the CESM model^79^. Abiotic oceanic sources of iodine (HOI and I_2_) are modelled according to the meteorology (temperature, winds, sea surface temperature and surface pressure) in CE 2010 based on MacDonald et al.^84^ parameterization. The authors would like to remark that a quantitative assessment of the role of iodine in aerosol radiative forcing (through its influence in NPF in the past) is yet not feasible mainly because there are still remaining chemical mechanistic gaps in our knowledge of the iodine particle formation and growth^4,87^.

## Supplementary information

Supplementary Information

## Data Availability

The ice core iodine data generated in this study have been deposited in the Zenodo database (10.5281/zenodo.5721369).

## Code availability

The software code for the CESM model is available from http://www.cesm.ucar.edu/models/.

## References

[1] Allan J (2015). Iodine observed in new particle formation events in the Arctic atmosphere during ACCACIA. Atmos. Chem. Phys..

[2] Roscoe HK (2015). Particles and iodine compounds in coastal Antarctica. J. Geophys. Res.: Atmos..

[3] Baccarini A (2020). Frequent new particle formation over the high Arctic pack ice by enhanced iodine emissions.. Nat. Commun..

[4] He X-C (2021). Role of iodine oxoacids in atmospheric aerosol nucleation. Science.

[5] O’Dowd CD (2002). Marine aerosol formation from biogenic iodine emissions. Nature.

[6] Sipilä M (2016). Molecular-scale evidence of aerosol particle formation via sequential addition of HIO_3_. Nature.

[7] Gómez Martín JC (2020). A gas-to-particle conversion mechanism helps to explain atmospheric particle formation through clustering of iodine oxides. Nat. Commun..

[8] Saiz-Lopez A (2007). Boundary layer halogens in coastal Antarctica. Science.

[9] Sherwen T (2016). Iodine’s impact on tropospheric oxidants: a global model study in GEOS-Chem. Atmos. Chem. Phys..

[10] Spolaor A (2013). Halogen species record Antarctic sea ice extent over glacial–interglacial periods. Atmos. Chem. Phys..

[11] Koenig TK (2020). Quantitative detection of iodine in the stratosphere. Proc. Natl Acad. Sci. USA.

[12] Saiz-Lopez A (2012). Estimating the climate significance of halogen-driven ozone loss in the tropical marine troposphere. Atmos. Chem. Phys..

[13] Hossaini R (2015). Efficiency of short-lived halogens at influencing climate through depletion of stratospheric ozone. Nat. Geosci..

[14] Sherwen T, Evans MJ, Carpenter LJ, Schmidt JA, Mickley LJ (2017). Halogen chemistry reduces tropospheric O 3 radiative forcing. Atmos. Chem. Phys..

[15] Iglesias-Suarez F (2020). Natural halogens buffer tropospheric ozone in a changing climate. Nat. Clim. Change.

[16] Legrand M (2018). Alpine ice evidence of a three-fold increase in atmospheric iodine deposition since 1950 in Europe due to increasing oceanic emissions. Proc. Natl Acad. Sci. USA.

[17] Cuevas CA (2018). Rapid increase in atmospheric iodine levels in the North Atlantic since the mid-20th century. Nat. Commun..

[18] Zhao X, Hou X, Zhou W (2019). Atmospheric iodine (127I and 129I) record in spruce tree rings in the Northeast Qinghai-Tibet Plateau. Environ. Sci. Technol..

[19] Corella JP (2019). Holocene atmospheric iodine evolution over the North Atlantic. Climate.

[20] Dahl-Jensen D (2013). Eemian interglacial reconstructed from a Greenland folded ice core. Nature.

[21] Rasmussen SO (2013). A first chronology for the North Greenland Eemian Ice Drilling (NEEM) ice core. Climate.

[22] Simonsen MF (2019). East Greenland ice core dust record reveals timing of Greenland ice sheet advance and retreat. Nat. Commun..

[23] Saiz-Lopez A (2012). Atmospheric chemistry of iodine. Chem. Rev..

[24] Fuge R, Johnson CC (2015). Iodine and human health, the role of environmental geochemistry and diet, a review. Appl. Geochem..

[25] Carpenter LJ (2013). Atmospheric iodine levels influenced by sea surface emissions of inorganic iodine. Nat. Geosci..

[26] Prados-Roman C (2015). A negative feedback between anthropogenic ozone pollution and enhanced ocean emissions of iodine. Atmos. Chem. Phys..

[27] Carpenter LJ (2021). Marine iodine emissions in a changing world. Proc. R. Soc. A: Math., Phys. Eng. Sci..

[28] Tham YJ (2021). Direct field evidence of autocatalytic iodine release from atmospheric aerosol. Proc. Natl Acad. Sci. USA.

[29] Saiz-Lopez A (2014). Iodine chemistry in the troposphere and its effect on ozone. Atmos. Chem. Phys..

[30] Gómez Martín JC (2021). Spatial and temporal variability of iodine in aerosol. J. Geophys. Res.: Atmos..

[31] Spolaor A (2016). Canadian Arctic sea ice reconstructed from bromine in the Greenland NEEM ice core. Sci. Rep..

[32] Maffezzoli N (2018). A 120 000-year record of sea ice in the North Atlantic?. Climate.

[33] Schüpbach S (2018). Greenland records of aerosol source and atmospheric lifetime changes from the Eemian to the Holocene. Nat. Commun..

[34] Simonsen MF (2018). Particle shape accounts for instrumental discrepancy in ice core dust size distributions. Climate.

[35] Hu A (2010). Influence of Bering Strait flow and North Atlantic circulation on glacial sea-level changes. Nat. Geosci..

[36] Kandiano ES, Bauch HA, Müller A (2004). Sea surface temperature variability in the North Atlantic during the last two glacial–interglacial cycles: comparison of faunal, oxygen isotopic, and Mg/Ca-derived records. Palaeogeogr., Palaeoclimatol., Palaeoecol..

[37] Bauch, H. A. & Kandiano, E. S. Evidence for early warming and cooling in North Atlantic surface waters during the last interglacial. *Paleoceanography* **22**, PA1201 (2007).

[38] Stein R, Fahl K, Gierz P, Niessen F, Lohmann G (2017). Arctic Ocean sea ice cover during the penultimate glacial and the last interglacial. Nat. Commun..

[39] Andrews J (1985). Land/ocean correlations during the last interglacial/glacial transition, Baffin Bay, northwestern North Atlantic: A review. Quat. Sci. Rev..

[40] Müller J (2011). Towards quantitative sea ice reconstructions in the northern North Atlantic: a combined biomarker and numerical modelling approach. Earth Planet. Sci. Lett..

[41] Otto-Bliesner BL (2013). How warm was the last interglacial? New model–data comparisons. Philosophical Transactions of the Royal Society A: Mathematical. Phys. Eng. Sci..

[42] McFarlin JM (2018). Pronounced summer warming in northwest Greenland during the Holocene and Last Interglacial. Proc. Natl Acad. Sci..

[43] Siddall M (2003). Sea-level fluctuations during the last glacial cycle. Nature.

[44] Dansgaard W (1993). Evidence for general instability of past climate from a 250-kyr ice-core record. Nature.

[45] Bond G (1993). Correlations between climate records from North Atlantic sediments and Greenland ice. Nature.

[46] Hoff U, Rasmussen TL, Stein R, Ezat MM, Fahl K (2016). Sea ice and millennial-scale climate variability in the Nordic seas 90 kyr ago to present. Nat. Commun..

[47] Rasmussen SO (2014). A stratigraphic framework for abrupt climatic changes during the Last Glacial period based on three synchronized Greenland ice-core records: refining and extending the INTIMATE event stratigraphy. Quat. Sci. Rev..

[48] Sadatzki H (2020). Rapid reductions and millennial-scale variability in Nordic Seas sea ice cover during abrupt glacial climate changes. Proc. Natl Acad. Sci. USA.

[49] Sadatzki H (2019). Sea ice variability in the southern Norwegian Sea during glacial Dansgaard-Oeschger climate cycles. Sci. Adv..

[50] Bory, A. M., Biscaye, P. E., Piotrowski, A. & Steffensen, J. P. Regional variability of ice core dust composition and provenance in Greenland. *Geochem. Geophys., Geosyst.* **4**, 1107 (2003).

[51] Svensson A, Biscaye PE, Grousset FE (2000). Characterization of late glacial continental dust in the Greenland Ice Core Project ice core. J. Geophys. Res.: Atmos..

[52] Jennings AE (2018). Baffin Bay paleoenvironments in the LGM and HS1: resolving the ice-shelf question. Mar. Geol..

[53] Müller J, Massé G, Stein R, Belt ST (2009). Variability of sea-ice conditions in the Fram Strait over the past 30,000 years. Nat. Geosci..

[54] Kolling HM, Stein R, Fahl K, Perner K, Moros M (2017). Short-term variability in late Holocene sea ice cover on the East Greenland Shelf and its driving mechanisms. Palaeogeogr. Palaeoclimatol. Palaeoecol..

[55] Müller J (2012). Holocene cooling culminates in sea ice oscillations in Fram Strait. Quat. Sci. Rev..

[56] Werner K (2016). Holocene sea subsurface and surface water masses in the Fram Strait–Comparisons of temperature and sea-ice reconstructions. Quat. Sci. Rev..

[57] Denton GH (2010). The last glacial termination. Science.

[58] Thompson WG, Goldstein SL (2006). A radiometric calibration of the SPECMAP timescale. Quat. Sci. Rev..

[59] Saiz-Lopez A, Blaszczak-Boxe CS, Carpenter L (2015). A mechanism for biologically induced iodine emissions from sea ice. Atmos. Chem. Phys..

[60] Saini J (2020). Holocene variability in sea ice and primary productivity in the northeastern Baffin Bay. arktos.

[61] Gibb OT, Steinhauer S, Fréchette B, de Vernal A, Hillaire-Marcel C (2015). Diachronous evolution of sea surface conditions in the Labrador Sea and Baffin Bay since the last deglaciation. Holocene.

[62] Slingo A (1990). Sensitivity of the Earth’s radiation budget to changes in low clouds. Nature.

[63] Gordon H (2017). Causes and importance of new particle formation in the present‐day and preindustrial atmospheres. J. Geophys. Res.: Atmospheres.

[64] Jansen E (2020). Past perspectives on the present era of abrupt Arctic climate change. Nat. Clim. Change.

[65] Kindler P (2014). Temperature reconstruction from 10 to 120 kyr b2k from the NGRIP ice core. Climate.

[66] Overland JE, Wang M (2013). When will the summer Arctic be nearly sea ice free?. Geophys. Res. Lett..

[67] Miller GH (2010). Arctic amplification: can the past constrain the future?. Quat. Sci. Rev..

[68] Volz A, Kley D (1988). Evaluation of the Montsouris series of ozone measurements made in the nineteenth century. Nature.

[69] Prados-Roman C (2015). Iodine oxide in the global marine boundary layer. Atmos. Chem. Phys..

[70] Winstrup M (2012). An automated approach for annual layer counting in ice cores. Clim.

[71] Vinther, B. M. et al. A synchronized dating of three Greenland ice cores throughout the Holocene. *J. Geophys. Res.: Atmos.* **111**, 10.1029/2005JD006921 (2006).

[72] Dansgaard W, Johnsen SJ (1969). A Flow model and a time scale for the ice core from camp century, Greenland. J. Glaciol..

[73] Vallelonga P (2017). Sea-ice-related halogen enrichment at Law Dome, coastal East Antarctica. Clim. Past..

[74] Andersen KK (2004). High-resolution record of Northern Hemisphere climate extending into the last interglacial period. Nature.

[75] Hansson ME (1994). The Renland ice core. A Northern Hemisphere record of aerosol composition over 120,000years. Tellus B.

[76] Hughes AG (2020). High-frequency climate variability in the Holocene from a coastal-dome ice core in east-central Greenland. Clim.

[77] Turekian, K. K. *Oceans*. 120 (Prentice-Hall, 1968).

[78] Hans Wedepohl K (1995). The composition of the continental crust. Geochimica et. Cosmochimica Acta.

[79] Lamarque JF (2012). CAM-chem: description and evaluation of interactive atmospheric chemistry in the Community Earth System Model. Geosci. Model Dev..

[80] Tilmes S (2016). Representation of the Community Earth System Model (CESM1) CAM4-chem within the Chemistry-Climate Model Initiative (CCMI). Geosci. Model Dev..

[81] Saiz-Lopez A (2015). Injection of iodine to the stratosphere. Geophys. Res. Lett..

[82] Fernandez RP, Salawitch RJ, Kinnison DE, Lamarque JF, Saiz-Lopez A (2014). Bromine partitioning in the tropical tropopause layer: implications for stratospheric injection. Atmos. Chem. Phys..

[83] Ordóñez C (2012). Bromine and iodine chemistry in a global chemistry-climate model: description and evaluation of very short-lived oceanic sources. Atmos. Chem. Phys..

[84] MacDonald S (2014). A laboratory characterisation of inorganic iodine emissions from the sea surface: dependence on oceanic variables and parameterisation for global modelling. Atmos. Chem. Phys..

[85] Meinshausen M (2011). The RCP greenhouse gas concentrations and their extensions from 1765 to 2300. Clim. Change.

[86] Rienecker MM (2011). MERRA: NASA’s modern-era retrospective analysis for research and applications. J. Clim..

[87] Martín JCG (2020). A gas-to-particle conversion mechanism helps to explain atmospheric particle formation through clustering of iodine oxides. Nat. Commun..

[88] Bates SL, Siddall M, Waelbroeck C (2014). Hydrographic variations in deep ocean temperature over the mid-Pleistocene transition. Quat. Sci. Rev..

[89] Laskar J (2004). A long-term numerical solution for the insolation quantities of the Earth. Astron. Astrophys..

