# Supplementary material for: Climate changes modulated the history of Arctic iodine during the Last Glacial Cycle
Source: Nat Commun. 2022 Jan 10;13:88. doi: 10.1038/s41467-021-27642-5 (PMC8748508; doi:10.1038/s41467-021-27642-5)
Supplement: Supplementary file 1 — Supplementary Information [file 41467_2021_27642_MOESM1_ESM.docx]

**Supplementary information**

**Climate changes modulated the history of Arctic iodine during the Last Glacial Cycle**

Juan Pablo Corella^1,2*^, Niccolo Maffezzoli^3,4.5^, Andrea Spolaor^4,5^, Paul Vallelonga^3^, Carlos A. Cuevas^1^, Federico Scoto^5,6^, Juliane Müller^7,8^, Bo Vinther^3^, Helle A. Kjær^3^, Giulio Cozzi^4,5^, Ross Edwards^9,10^, Carlo Barbante^4,5^ and Alfonso Saiz-Lopez^1*^

^1^Department of Atmospheric Chemistry and Climate, Institute of Physical Chemistry Rocasolano, CSIC, Serrano 119, 28006 Madrid, Spain

^2^Present address: CIEMAT, Environmental Department, Av. Complutense 40, 28040, Madrid, Spain

^3^Physics of Ice Climate and Earth, Niels Bohr Institute, University of Copenhagen, Tagensvej 16, Copenhagen N 2200, Denmark

^4^Institute of Polar Sciences, CNR- ISP, Via Torino 155, 30170 Venice, Italy

^5^Ca’ Foscari University of Venice, Department of Environmental Sciences, Informatics and Statistics, Via Torino 155, 30172 Venice, Italy

^6^Institute of Atmospheric Sciences and Climate, ISAC-CNR. S.P Lecce-Monteroni km1.2, 73100 Lecce, Italy

^7^Alfred Wegener Institute, Helmholtz Center for Polar and Marine Research, Am Alten Hafen 26, 27568 Bremerhaven, Germany

^8^MARUM Research Faculty, University of Bremen, Leo­bener Strasse 8, 28359 Bre­men, Ger­many

^9^Physics and Astronomy, Curtin University, Kent St, Bentley WA 6102, Australia

^10^Department of Civil and Environmental Engineering, UW-Madison, Madison, WI 53706, USA


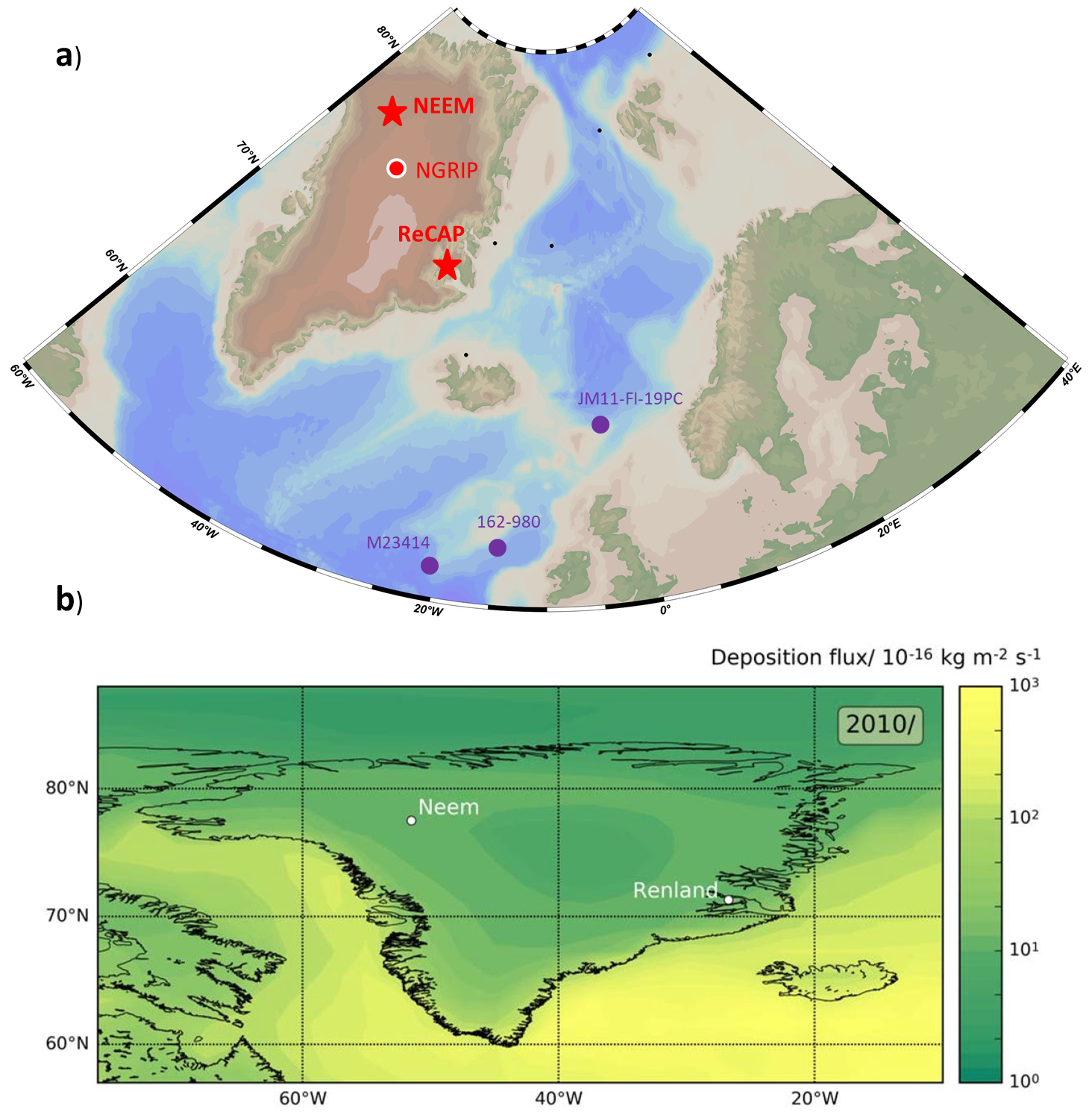


**Supplementary Figure 1:** **Present-day (CE 2010) iodine depositional fluxes over Greenland**. Modelled mean annual reactive iodine deposition fluxes over the Greenland continental area using a CAM-Chem model


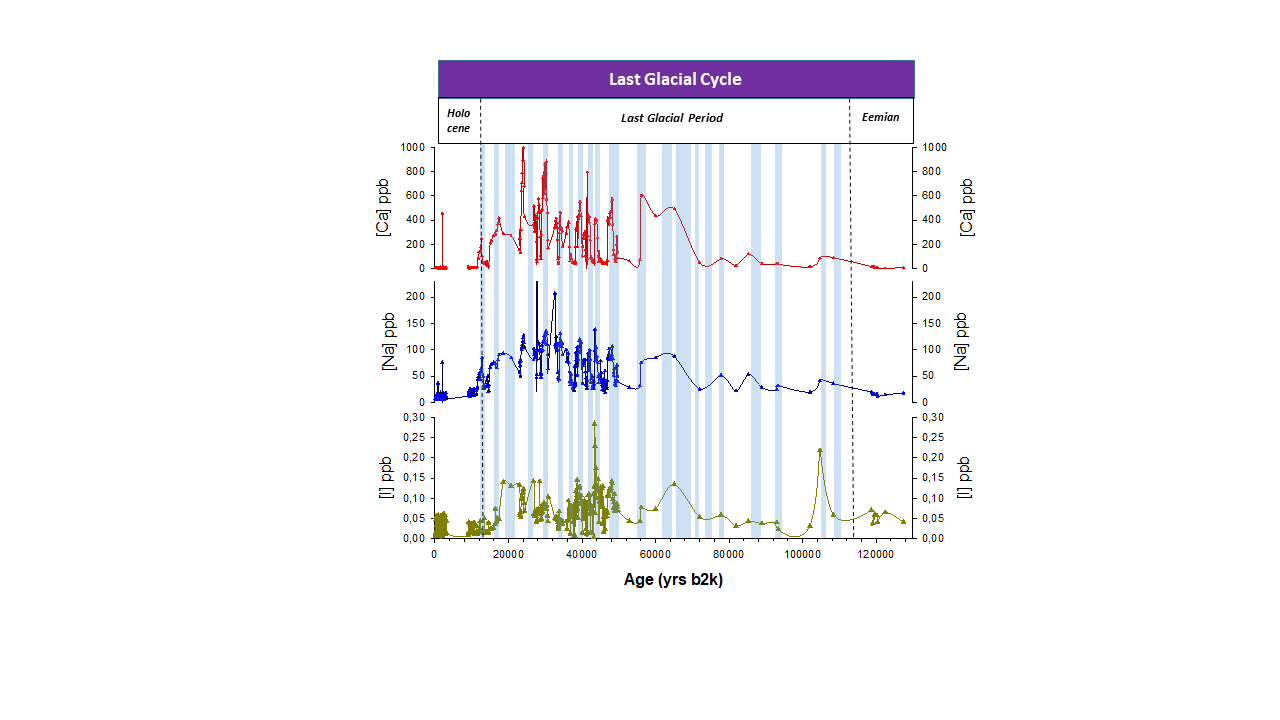


**Supplementary Figure 2:** **Iodine levels evolution in NEEM ice core during the LGC.** From bottom to top; Iodine (I), sodium (Na) and calcium (Ca) concentrations. Blue bands indicate cold stadial periods.


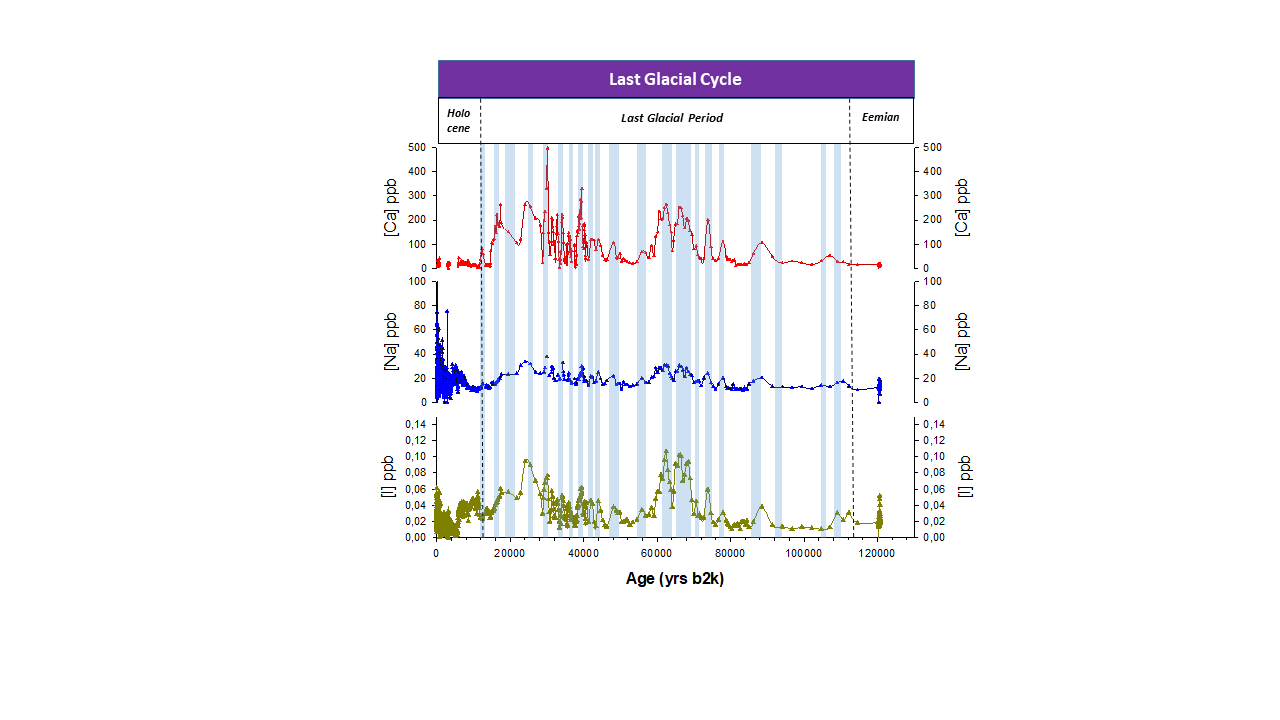


**Supplementary Figure 3:** **Iodine levels evolution in ReCAP ice core during the LGC.** From bottom to top; Iodine (I), sodium (Na) and calcium (Ca) concentrations. Blue bands indicate cold stadial periods.

** Supplementary Figure 4:** **Iodine levels evolution in NEEM ice core between 34-42 yrs b2k (GS6-GS11).** From bottom to top; Iodine (I), sodium (Na) and calcium (Ca) concentrations.
